# Identifying geopolitical event precursors using attention-based LSTMs

**DOI:** 10.3389/frai.2022.893875

**Published:** 2022-10-31

**Authors:** K. S. M. Tozammel Hossain, Hrayr Harutyunyan, Yue Ning, Brendan Kennedy, Naren Ramakrishnan, Aram Galstyan

**Affiliations:** ^1^Institute for Data Science & Informatics, University of Missouri, Columbia, MO, United States; ^2^Information Sciences Institute, University of Southern California, Marina del Rey, CA, United States; ^3^Department of Computer Science, Stevens Institute of Technology, Hobken, NJ, United States; ^4^Department of Computer Science, University of Southern California, Los Angeles, CA, United States; ^5^Department of Computer Science, Virginia Tech Research Center-Arlington, Virginia Tech, Arlington, VA, United States

**Keywords:** event forecasting, event precursors, social unrest modeling, attention-method, deep learning, long short-term memory (LSTM)

## Abstract

Forecasting societal events such as civil unrest, mass protests, and violent conflicts is a challenging problem with several important real-world applications in planning and policy making. While traditional forecasting approaches have typically relied on historical time series for generating such forecasts, recent research has focused on using open source surrogate data for more accurate and timely forecasts. Furthermore, leveraging such data can also help to identify precursors of those events that can be used to gain insights into the generated forecasts. The key challenge is to develop a unified framework for forecasting and precursor identification that can deal with missing historical data. Other challenges include sufficient flexibility in handling different types of events and providing interpretable representations of identified precursors. Although existing methods exhibit promising performance for predictive modeling in event detection, these models do not adequately address the above challenges. Here, we propose a unified framework based on an attention-based long short-term memory (LSTM) model to simultaneously forecast events with sequential text datasets as well as identify precursors at different granularity such as documents and document excerpts. The key idea is to leverage word context in sequential and time-stamped documents such as news articles and blogs for learning a rich set of precursors. We validate the proposed framework by conducting extensive experiments with two real-world datasets—military action and violent conflicts in the Middle East and mass protests in Latin America. Our results show that overall, the proposed approach generates more accurate forecasts compared to the existing state-of-the-art methods, while at the same time producing a rich set of precursors for the forecasted events.

## 1. Introduction

Forecasting spatiotemporal political events, including protests, crimes, riots, and inter-state interactions and conflicts, has garnered lots of attention in recent years. A trend in forecasting such events is to exploit surrogate data sources such as social media and news feeds (Muthiah et al., 2015), social network information (Chen and Neill, 2014; Cadena et al., 2015), and mobile communication data (Lu et al., 2012) with machine learning techniques. In this paper, we study the problem of forecasting protests, political conflicts, and violent events along with providing supporting evidence (precursors) for the forecasts using text documents from online media.

Early event warning systems integrate domain knowledge, information extraction algorithms, and statistical prediction models to produce warnings. It is critical that the “visibility" of these warnings matches the quality of the actual, produced warnings. By visibility, we are referring to the requirement that event forecasts provide evidence accompanying each forecast. To this challenging objective, previous studies (Ning et al., 2016, 2018; Xue et al., 2018) adopt multi-instance learning to cast the event forecasting problem into a classification task and identify “precursor" news articles for the events of interest. These studies suffer from two limitations: 1) Sensitivity to the bags with fewer instances and noisy data that do not describe a relation at all. 2) Difficulty in handling precursor representations at different levels such as document or document excerpts levels.

The term *precursor* merits further discussion. Historically, event forecasting has, by necessity, occurred at a highly aggregated level. The reason is that most approaches to longitudinal event analysis, up until the past several decades, occurred at the country-year level. As event data have become increasingly disaggregated (e.g., city-day level) the possibility of forecasting individual events has become more realizable. With such forecasts of individual events, the analysts and policy makers would need to know not only with what confidence these forecasts are made but *why* they are made. In this context, we conduct our research: we seek to empirically link event forecasts to pieces of evidence without specifying the nature of evidence beforehand.

We present—EPIAL—**E**vent **P**recursor **I**dentification using **A**ttention-based **L**STM for event forecasting and precursor identification. Long Short-Term Memory (LSTM) networks have shown success in modeling long-term dependencies in sequential data. Attention mechanisms are studied to learn which subsets of the sequential units are useful. Intuitively, the design of this model allows us to capture two critical elements of an event forecast: (1) the temporal relationship between documents and events; and (2) which documents have the greatest impact on event outcomes.

We evaluate our models using two datasets: Military Action and Non-State Actor (MANSA) events in the Middle East and North Africa (MENA) region, and civil unrest events in Latin American countries. We collect a large set of Arabic news articles for MANSA event prediction, and news articles published in Spanish and Portuguese over the Latin American countries.

**Figure 1:**
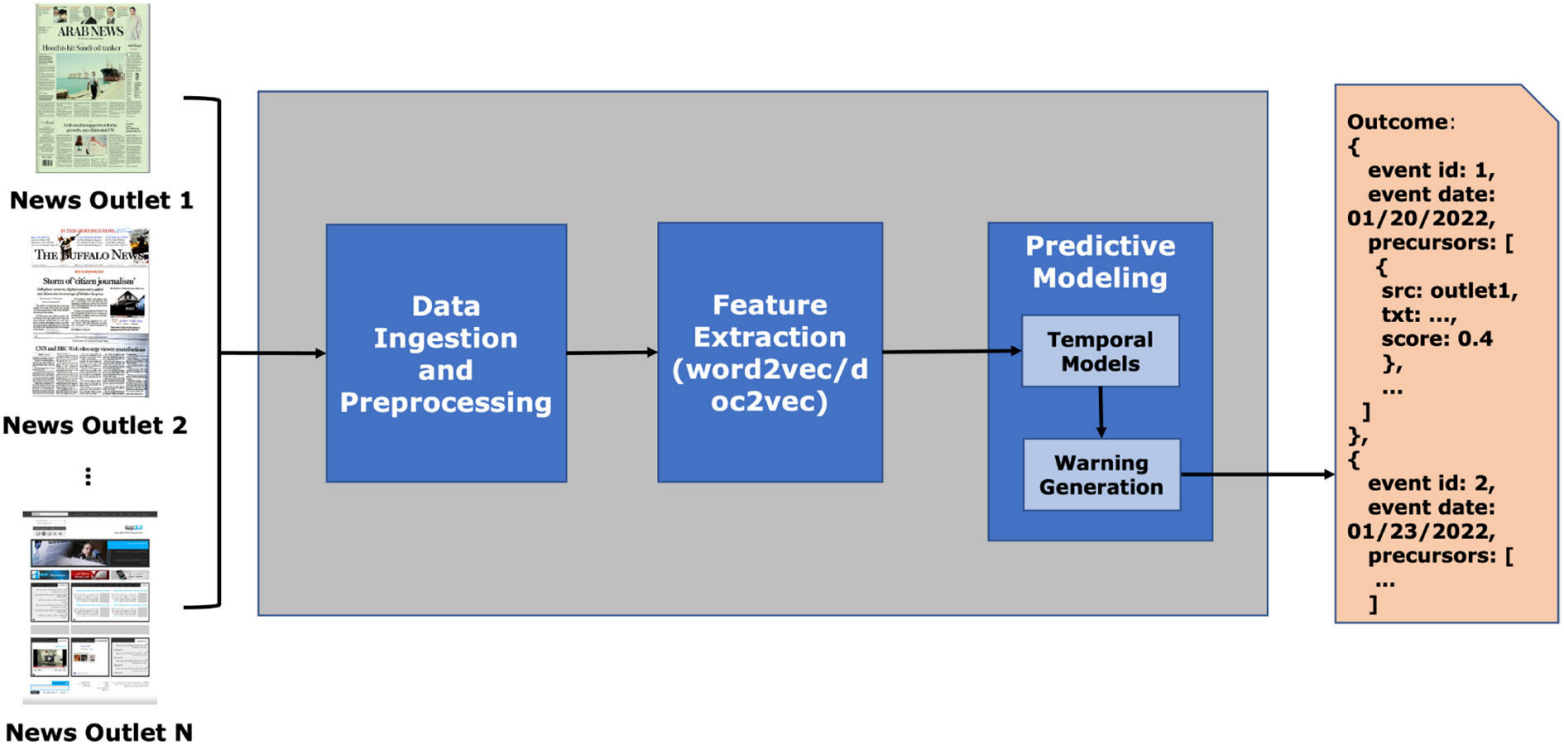
A framework for anticipating geopolitical events with precursors.

The framework for this study comprises three modules (refer to Figure 1). The Data Ingestion and Preprocessing module fetches data from Arabia Inform and performs basic prepossessing. The Feature Extraction module transforms the article text to features required by the Predictive Modeling module. Within the Predictive Modeling module, we implement various temporal predictive models, which forecast events and identify precursors (i.e., articles or article snippets). The Warning Generation submodule takes these forecasts and precursors for producing alerts.

Our key contributions are as follows:

- We propose a unified, deep-learning based approach for forecasting events with sequential data as input, which automatically identifies multiple levels of precursors using an attention mechanism. The framework requires minimal feature engineering, by leveraging distributed document representation.
- We demonstrate that the proposed method is scalable and robust with respect to different event categories in multiple geographical locations, and handles missing historical data organically without any manual feature tuning.
- We demonstrate the efficacy of our method by conducting extensive experiments on two events datasets: a) Middle East MANSA events and b) Latin American protest events. Our results suggest that the proposed framework produces better predictive performance compared to several state-of-the-art methods while producing a richness of precursors for the forecasted events.

The rest of this paper is organized as follows: In Section 2, we discuss the connection of the proposed approaches to related work. Section 3 presents the details of the proposed model. The experimental results are presented in Section 4. Section 5 concludes the paper.

## 2. Related study

In this section, we discuss research pertinent to our study. Over the last decade, there has been a significant interest in gaining insights into societal events, which are of two types: a) offline (e.g., protest, organized crime, and epidemic) and b) online (e.g., activism, petitions, and rumor) (Deng and Ning, 2021; Zhao, 2021). The modeling of such societal events falls under two broad categories–a) retrospective studies such as event detection (Yang et al., 1998) and summarization (Chakrabarti and Punera, 2011) and b) event forecasting (Zhao et al., 2018), i.e., anticipating future events. A variant of the event forecasting problem is to infer precursors to forecasts (Ning et al., 2016, 2018; Xue et al., 2018). From the perspective of datasets, some studies solely use historical events for modeling events, whereas others use both historical events and external datasets (e.g., mass media). This paper focuses on generating forecasts along with precursors for offline events, specifically for civil unrest and violent conflicts caused by the military (e.g., Syrian Military) and non-state actors (e.g., ISIS), and the models exploit event history and news media. Below we present recent studies on forecasting civil unrest and geopolitical events as well as studies on precursor identification.

### 2.1. Forecasting civil unrest and geopolitical events

The prediction of violent political and civil unrest events is an interdisciplinary field. Several machine learning methods, supervised and unsupervised, have been developed to predict such events. The linear regression models use simple features to predict the occurrence time of future events (O'Connor et al., 2010; Bollen et al., 2011; He et al., 2013; Arias et al., 2014). More advanced techniques use features such as topic-related keywords as input to support vector machines, LASSO, and multi-task learning approaches (Ritterman et al., 2009; Wang et al., 2012). Ramakrishnan et al. (2014) designed a framework (EMBERS) for predicting civil unrest events in different locations by using a wide combination of models with heterogeneous input sources, including social media, news articles, and satellite images. Other efforts include (Cadena et al., 2015), which learns associations between relevant activity cascades on social media with real-world protest events, and (Boecking et al., 2015), which represents social media posts as latent text representations, and use these to forecast events surrounding the Egyptian Revolution of 2011.

Schrodt et al. (2013) provide a review of event extraction, event data standards, and statistical methods for predicting political violence, including Hidden Markov Models, time-series auto-regression, classifier-based forecasting, and graph and network-based approaches. Base-rate methods—which only use event history —for political violence include (Zammit-Mangion et al., 2012), which employs a log Gaussian Cox process to model military event data from the WikiLeaks Afghanistan data, and (Raghavan et al., 2013; Hossain et al., 2018), which models activity levels of terrorist groups with an HMM.

Zhao et al. (2015b) combine multi-task learning and dynamic features from social networks for spatial-temporal event forecasting. Generative models have also been used in Zhao et al. (2015a) to jointly model the temporal evolution in semantics and geographical burstiness within social media content. Laxman et al. (2008) designed a generative model for categorical event prediction in event streams using frequent episodes.

### 2.2. Event forecasting with external text corpora

To increase available information to predictive models in spatial and temporal contexts, recent research has attempted to forecast events in real-time using text corpora, such as news documents and social media posts, as well as other open source indicators. Text-based methods for forecasting events are highlighted by Ning et al. (2016), which identifies news articles as precursor indicators for protest events with a multi-instance learning model. This model focuses on jointly forecasting events and identifying evidence for such forecasts, an element that is critical in the application of event forecasts. By extracting event sequences from the text and learning causal associations through knowledge-base inference, several models are proposed (Radinsky et al., 2012a,b; Radinsky and Horvitz, 2013) to forecast events of disease outbreaks, deaths, and riots. Events are learned from text by clustering story-lines according to their textual and semantic entity similarity. Predictions are made in these methods by pattern-matching known event chains with test data. A recent trend is to build a dynamic knowledge graph of actors and their interactions and exploit the graph for predicting future events (Trivedi et al., 2017). Mueller and Rauh (2017) proposed to predict political violence with changes in news topics shared at the country-year level over a period of time in the twentieth Century. Muthiah et al. (2015) studied forward-referencing planned events modeling from news sources and social media.

### 2.3. Precursor identification

Although there are many methods for forecasting events, few existing approaches provide evidence and interpretive analysis as support for event forecasting. Hence, identifying precursors for significant events is an interesting topic and has recently gained much interest. Rong et al. (2015) developed a combinational mixed Poisson process (CMPP) model to learn about social, external and intrinsic, influence in social networks. A more recent method of precursor identification is proposed by Ning et al. (2016). In this method, a nested multi-instance framework is presented to forecast civil unrest events and detect precursor news articles for these events. Xue et al. (2018) proposed a method combining multi-instance and LSTM networks to identify precursors. In these methods, the multi-instance framework is used for identifying precursors, whereas we propose an attention mechanism for inferring precursors. The methods proposed in this paper can be viewed as complementary to the prior work discussed above, casting the temporal event forecasting and precursor discovery problems into a sequence learning framework.

There are recent methods that use both temporal and spatial features to identify precursors (Ning et al., 2018). As spatial features are missing in our external dataset, we focus only on the temporal aspect of the text data for this study.

### 2.4. Attention-based models

Long Short-Term Memory (LSTM) Recurrent Neural Networks (Hochreiter and Schmidhuber, 1997) have been shown to be effective for many tasks (Sutskever et al., 2014; Gregor et al., 2015; Vinyals et al., 2015; Wang and Jiang, 2016). The ability to learn long term dependencies, along with attention mechanisms gives LSTM superior performance in many natural language processing tasks: machine translation, sentiment analysis, question answering, etc. The attention mechanism selects a subset from a sequence of inputs by generating attention scores for each element of the sequence, where these scores are calculated conditioned on the internal state of the model and external inputs. The attention mechanism makes the model more interpretable and gives the model the ability to ignore irrelevant information. This is highly effective in question answering systems when the model learns to attend to parts of the text which are related to the answer to the question (Hermann et al., 2015; Wang and Jiang, 2016; Wang et al., 2017). In machine translation, to generate the next word from the translation sentence, the model uses an attention mechanism to attend to the part of the source sentence, which is related to the translation of the current word (Bahdanau et al., 2014).

## 3. Methods

In this section, we first formulate the problem. We then present EPIAL and its variant.

### 3.1. Problem formulation

**Figure 2:**
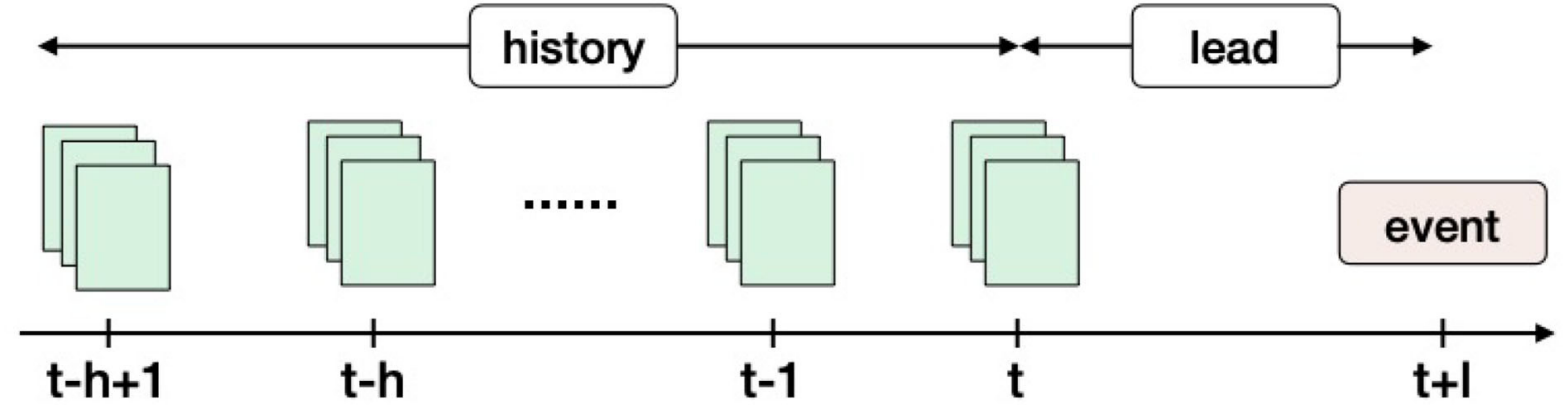
Event precursors identification overview. Here, *h* denotes the number of timestamps in the event history and *l* is the lead time. An event history could contain one or more external data sources (e.g., news articles, tweets, etc). The key idea in this framework is to anticipate an event at time point *t* + *l* using the history in the time window [*t* − *h* + 1, *t*]. This problem setting ensures a minimal lead time (*l*) with each prediction.

Given an ordered sequence of documents within a time window [*t* − *h* + 1, *t*], we aim to predict whether an event of interest will happen at time point *t* + *l*, where *h* is the history length and *l* is the lead time (refer to Figure 2). In the training data, we are given a set of such sequences followed by event occurrences for each of them.

Let *y* be a binary variable denoting the occurrence of an event, and $C^{\left(t\right)}=\left\{D_{1}^{\left(t\right)},D_{2}^{\left(t\right)},\ldots ,D_{k_{t}}^{\left(t\right)}\right\}$ be the set of news articles on day *t*, where *k*_*t*_ is the number of articles of day *t*. Each document *D* is a list of tokens, *D* = (*w*_1_, *w*_2_, …, *w*_*m*_), where *m* is the number of tokens. Given ${\left\{\left(C_{i}^{\left(t\right)},y_{i}\right)\right\}}_{i=1}^{n}$, the proposed models seek to predict *y*_*i*_ as well as identify documents or document excerpts in ${\left\{C^{\left(t\right)}\right\}}_{t=1}^{h}$ that are precursors to *y*_*i*_. Here, *y*_*i*_ denotes the occurrence of the *i*-th event.

The EPIAL can be evaluated using the whole texts of documents as well as only using excerpts, which could be defined as the context of keywords.

### 3.2. Proposed models

We propose EPIAL and its variant EPIAL-light, both based on LSTM recurrent neural networks (Hochreiter and Schmidhuber, 1997). LSTM is a type of recurrent neural network designed to capture long-term dependencies in sequential data. A simple LSTM unit consists of a cell unit and three gates—input (a.k.a remember), forget, and output. The LSTM takes a sequence ${\left\{x_{t}\right\}}_{t=1}^{T}$ as its input and provides a sequence of hidden state vectors, ${\left\{h_{t}\right\}}_{t=1}^{T}$ using the following equations:


$$\begin{array}{ll} i_{t} & =\sigma \left(x_{t}W_{xi}+h_{t-1}W_{hi}\right)\\ f_{t} & =\sigma \left(x_{t}W_{xf}+h_{t-1}W_{hf}\right)\\ c_{t} & =f_{t}⊙c_{t-1}+i_{t}⊙\tanh\left(x_{t}W_{xc}+h_{t-1}W_{hc}+b_{c}\right)\\ o_{t} & =\sigma \left(x_{t}W_{xo}+h_{t-1}W_{ho}+b_{o}\right)\\ h_{t} & =o_{t}⊙\tanh\left(c_{t}\right) \end{array}$$

Here, σ (sigmoid) and tanh functions are applied element-wise. The matrices W_*_ and vectors b_*_ are the trainable parameters of the LSTM. Here, we use many-to-one LSTM layers, i.e., we use the last output, *h*_*T*_. The rest of this paper uses *h*_*T*_ = LSTM(*x*_*t*_) as a shorthand for calculating *h*_*T*_ from *x*_*t*_ using an LSTM layer. Note that we could have used the GRU unit instead of the LSTM unit as they have similar functionality, but we preferred LSTM for its effectiveness (Chung et al., 2014). The methods we propose take word vectors of documents as input. We train word vectors on the training data using the GloVe algorithm (Pennington et al., 2014). We do not use pre-trained word vectors because they are trained in a general context and can miss important aspects of our problem.

**Figure 3:**
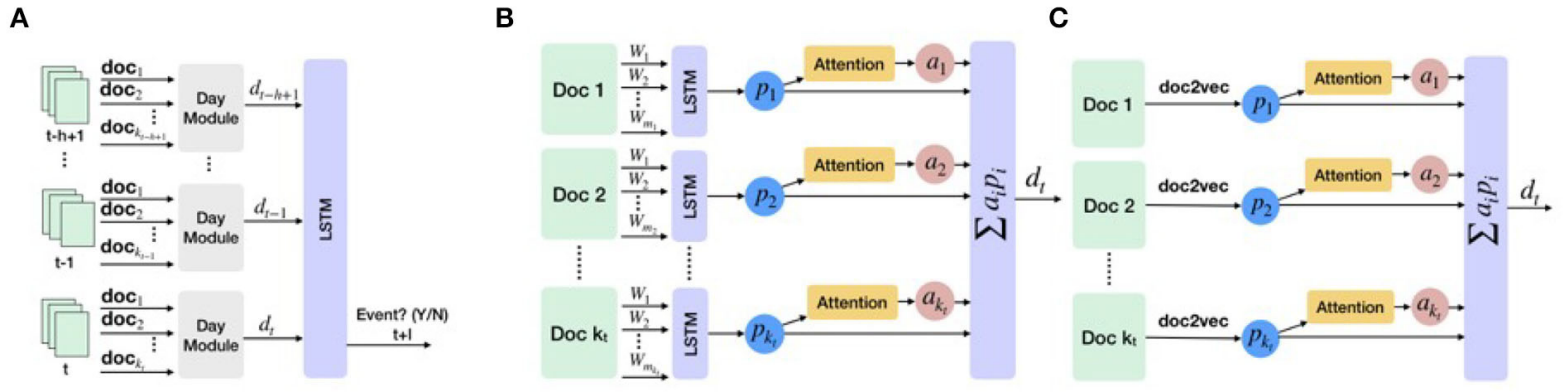
Overview of the proposed framework using attention-based LSTM. **(A)** The overall structure of the attention-based LSTM model. **(B)** Day module in EPIAL learns representation for each document and its attention score. **(C)** Day module in EPIAL-light replaces the first LSTM layer of EPIAL with document representation estimated using doc2vec.

The overall architecture of EPIAL is illustrated in Figure 3A. The key idea is that the day module learns representations of documents and their attention scores, and combines these representations to provide a daily representation, *d*_*t*_. Given ${\left\{d_{i}\right\}}_{i=t-h+1}^{t}$ from the daily module, the output LSTM layer takes the sequence as input and outputs the probability that an event will happen: $\hat{y}=\text{LSTM}\left(d\right)$. We use the sigmoid activation function in this output LSTM layer.

In the day-module of EPIAL (refer to Figure 3B), an LSTM layer is used to construct representation *p*_*i*_ for each document in a day. We use weight sharing across the LSTM layers of different days. Given document representations, we use an additive attention function (Bahdanau et al., 2014), which is a feed-forward neural network with two layers to compute compatibility scores of documents. Given the representations (*p*_1_, *p*_2_, …, *p*_*k*_) of documents of a day, attention scores are computed in the following way:


$$\begin{array}{l} s_{i}=W^{\left(2\right)}\text{tanh}\left(W^{\left(1\right)}p_{i}+b^{\left(1\right)}\right) \end{array}$$

where *W*^(1)^, *b*^(1)^, and *W*^(2)^ are the parameters of the attention network. These parameters are shared with parameters of attention networks of other days. We use soft attention, *a*_*i*_ = softmax(*s*)_*i*_, to estimate the document's probability of being a precursor. The daily representation *d*_*t*_ of the documents is estimated as, $d_{t}=\sum_{i=1}^{k}a_{i}p_{i}$.

In the day-module of EPIAL-light (refer to Figure 3C), we aim to identify precursors only at the document level. Instead of using an LSTM layer for learning document representations, we learn document representations for the entire corpus with the doc2vec method (Le and Mikolov, 2014), and use these representations for computing daily representation *d*_*t*_. As document vectors are learned prior to the training of EPIAL-light, this model runs faster than EPIAL, but it may not capture the complex relationship between document-level excerpts and events. The key difference between EPIAL and EPIAL-light is that EPIAL uses word-level embeddings as input, and EPIAL-light uses document-level embeddings. The EPIAL method can identify precursors at the word, sentence, paragraph, and document levels, whereas EPIAL-light discovers precursors at the document level.

Following Pham et al. (2014), we apply dropout on non-recurrent connections between the LSTM layers and the outputs. We adopt cross-entropy as our loss function: $L\left(y,\hat{y}\right)=-\left(y\log\hat{y}+\left(1-y\right)\log\left(1-\hat{y}\right)\right)$.

## 4. Experiments

In this section, we present a detailed evaluation of real-world datasets and the reasoning behind the results of our method. We design experiments to address the following questions.

1. How does the method compare against existing methods in terms of predicting events? (see Section 4.4)
2. How is the model's performance influenced by various parameters such as history length and lead time? (see Section 4.5)
3. Does the model identify meaningful precursors? How well do the precursors make a story? (see Section 4.6)

### 4.1. Datasets

For the training and evaluation of our methods, we use two real-world event datasets.

#### 4.1.1. Mid-East MANSA dataset

**Ground truth:** The ground truth information about MANSA events, called the gold standard report (GSR), is exclusively provided by the Center for Analytics at New Heaven (IARPA, 2018). The GSR is a manually created list of MANSA events by domain experts. Each event in the dataset has 13 different attributes: actor, actor status, approximate location, causalities, country, earliest reported date, encoding comment, event date, event id, event subtype, event type, first reported link, gold standard source link, latitude, longitude, news source, other links, revision date, state, target, target name, target status. While much care had been taken for addressing attribution and duplication problems in the manual event documentation step, we also remove any duplicates in preprocessing steps using these attributes.

The MANSA ground truth dataset contains violent geopolitical events that occurred between May 2015 to Oct 2017. This dataset covers several countries in the Middle East, such as Syria, Iraq, Egypt, Lebanon, Saudi Arabia, Yemen, and Jordan. For evaluating our model, we focus on cities in Syria and Iraq as the events in other countries are very sparse. We primarily focus on three cities: Baghdad, Damascus, and Ar Raqqah. As the events in Syria and Iraq are abundant during the event period, the training set (refer to Section 4.3) becomes imbalanced if we focus on the entire country. These selected GSR events are used for validation of our forecasting algorithm.

**External dataset:** For external features, we use news surrogate data which is generated from Arabic news articles from MENA countries provided by Arabia Inform (Inform, 2017). Curators of the ground truth provided a set of 29 keywords (including “terrorist,” “bomb,” and “military”) that they use to search for ground truth news articles. We use the same set of keywords to filter out candidate news articles for generating forecasts: we select an article if it contains at least one of these keywords. This corpus of news articles contains the newspaper name, the publication date, article text, and city names (if mentioned). We apply our second filter to create document sets for each of these three cities. We performed standard preprocessing of the text (light stemming, stop-word removal). As the news articles in our dataset are predominantly in Arabic (90%), we perform light stemming, as Arabic is a highly inflecting language (Larkey et al., 2007) and the development of a proper Arabic lemmatizer is still an active area of research.

Table 1 shows counts of events and news articles related to Baghdad, Damascus, and Ar Raqqah.

**Table 1:** Datasets of MANSA events and LA protests.

**City**	**Events**	**Articles**	**Country**	**Events**	**Articles**	**City**	**Events**	**Articles**
Baghdad	656	25,9132	Argentina	921	10,386	Buenos Aires	391	10,386
Damascus	796	23,6632	Brazil	2073	27,133	Brasilia	257	27,133
Raqqah	856	67,746	Colombia	871	8,386	Medellin	213	8,386

For LA protest datasets, We also create data subsets for Buenos Aires (Argentina), Brasilia (Brazil), and Medellin (Colombia).

#### 4.1.2. LA protest dataset

**Ground truth:** The Latin American protest dataset contains protest events that MITRE (Ramakrishnan et al., 2014) collected over several countries in Latin America from July 2012 to December 2014. A labeled GSR event provides information about the geographical location at the city level, date, type, and population of a civil unrest news report extracted from the most influential newspaper outlets within the country of interest. These GSR reports are the target events that are used for validation of our forecasting algorithm.

**External dataset:** For forecasting events and precursor identification was performed on news articles collected from around 6,000 news agencies between July 2012 and December 2014 across several countries in South America, including Argentina, Brazil, and Colombia. For Argentina and Colombia, the input news articles were primarily in Spanish and for Brazil, the news articles were in Portuguese. Each news article and event report contains meta data such as *publication date, location (country, state, city), text description, and label for protest event (yes/no)*. We focus on country-level and city-level events and select three cities: Brasilia (Brazil), Buenos Aires (Argentina), and Medellin (Colombia).

Table 1 shows counts of events and news articles related to Argentina, Brazil, and Colombia.

### 4.2. Comparison methods

We compare the proposed models to the following approaches.

- **Baseline**: Predicts events respecting the training set's class distribution.
- **Support Vector Machine (SVM)** (Cortes and Vapnik, 1995): In our event forecasting setting SVM method essentially takes frequency counts of predefined keywords over the history as features with the occurrence of an event as the label.
- **Relaxed Multi-Instance Learning (MIL)** (Wang et al., 2015): We form the historical news articles before an event of interest into a bag. The model estimates the probability of each instance in a bag using a logistic function. Then we use the average of instance-level probabilities to model the probabilities for bags.
- **Nested Multi-Instance Learning (nMIL)** (Ning et al., 2016): This approach applies a nested level of multi-instance learning for the event forecasting problem. It estimates the probability of each historical news article with doc2vec representations.

### 4.3. Experimental setup

For creating a training instance for geolocation, we label a day based on the occurrences of events (binary outcome). We then choose a lead time (*l*) and history length (*h*) and identify the dates to look at for articles.

1. *Positive Instances:* for a ground truth event at time point *t*, we extract news articles that occurred over *h* days from the time point (*t* − *l* − *h* + 1) to *t* − *l*.
2. *Negative Instances:* identify a day with no ground truth event reported and extract news articles that occurred over *h* days from the time point (*t* − *l* − *h* + 1) to *t* − *l*.

We allow days with missing documents while creating instances. We exclude datasets that are highly imbalanced in terms of positive and negative instances (e.g., Aleppo, Mosul, Homs, Syria, and Iraq). While analyzing conflicts in Syria and Iraq, we found that the military and non-state actor-based events happened centered around some cities almost daily between 2015 and 2017 (this duration marked the rise of ISIS and other rebel groups). Hence, the datasets on entire Syria, Iraq, and some cities, including Aleppo, Mosul, Idlib, and Homs, are highly imbalanced. To include these highly imbalanced datasets, we could apply up-sampling methods, but developing a model for generating reliable synthetic ground truth data for societal events is challenging and an active area of research. We plan to explore this direction in the future.

We evaluate the predictive capability of our proposed model against four methods (refer to Section 4.2) in terms of precision, recall, accuracy, F1-score, and area under the ROC curve (AUC). For each of these methods, we create three splits of data with shuffling and stratification so that train (85%) and test sets (15%) have a proportional representation of class labels. For EPIAL and EPIAL-light, we use 15% of train data for validation. We use three splits, which is equivalent to 3-fold cross-validation. We compared the performance of EPIAL and EPIAL-light with 3-splits and 5-splits and did not find significant differences. As EPIAL takes a considerable amount of computing time, we performed 3-splits of data to reduce overall computing time. To be consistent, we use a 3-splits evaluation for other models.

Without loss of generality, we use document excerpts instead of the entire document for faster training. We first organize a set of domain-related keywords. Given a *context window* (*c*) parameter, we create the context of each predefined keyword present in the document by extracting *c* words from the left and the right of the keyword. We then merge the contexts into excerpts. For the MANSA datasets, we use 29 keywords along with names of actors, countries, and cities in the region. For the LA dataset, we use a list of 2,800 manually curated keywords.

We develop our model in Keras with Tensorflow backend (the code and dataset will be published on GitHub). We optimize loss function with Adam (Kingma and Ba, 2014) optimizer with parameters α = 0.001 and β_1_ = 0.9. We train word vectors for EPIAL of sizes 50, 100, 150, and 200 with Glove (Pennington et al., 2014). For EPIAL-light we use the Gensim doc2vec module (Gensim, 2018) with vector size 50, 100, 150, and 200. The number of LSTM units is 32 by default, but we also tested our method with 16, 64, and 128 units. We also tested our model with various input dropout rates such as 0.0, 0.1, 0.2, and 0.3, and batch size 1, 2, 4, 8, 16, and 32. We use a validation set for model selection. By default, we run each EPIAL and EPIAL-light for 75 epochs, and we estimate validation loss over epochs. We identify a strip of epochs where validation loss is stable, and we select the model at an epoch with minimal validation loss (refer to Supplementary Figure S2).

### 4.4. Predictive performance of the models

We compare the predictive performance of EPIAL and EPIAL-light with four different models (refer to Section 4.2) ranging from simpler (Baseline and SVM) to more sophisticated ones (MIL and nMIL) for different event types in multiple geolocation settings. More specifically, we predict events at the city level for the MANSA dataset, whereas for the LA protest dataset we predict both at the city and country levels. In most of the datasets, we observe that EPIAL and EPIAL-light outperform other methods including nMIL in terms of four different scores: precision, recall, F1-score, accuracy, and AUC. The EPIAL and EPIAL-light methods secure the best F1-score in eight out of nine, the best accuracy in all of the nine datasets, and the best AUC score in seven out of nine datasets (refer to Table 2). The EPIAL and EPIAL-light models seemed to show better performance at the country and city levels. We also observe that EPIAL-light performs better than the nMIL method for most of the datasets. For Baghdad EPIAL and EPIAL-light do not achieve high F1-scores, which could be related to the quality of the documents in this dataset. We do not evaluate our method with country level MANSA events as the training datasets are imbalanced due to the high density of events at the country level.

**Table 2:** Comparison between six models in terms of forecasting performance.

**Method (→)** **City (↓)**	**Baseline**	**SVM**	**MIL**	**nMIL**	**EPIAL-light**	**EPIAL**
Ar Raqqah	0.68, 0.73, 0.70, 0.62, 0.58	0.69, 0.87, 0.77, 0.68, 0.63	**0.80**, 0.43, 0.46, 0.52, 0.55	0.60, 0.73, 0.65, 0.60, 0.55	0.76, 0.84, 0.80, 0.73, 0.75	0.79, **0.91**, **0.85**, **0.79**, **0.79**
Damascus	0.54, 0.51, 0.52, 0.50, 0.50	0.61, 0.72, 0.66, 0.60, 0.59	0.50, 0.37, 0.42, 0.46, 0.47	0.56, 0.73, 0.59, 0.55, 0.60	0.69, 0.77, 0.73, 0.68, 0.68	**0.72**, **0.83**, **0.77**, **0.73**, **0.78**
Baghdad	0.41, 0.42, 0.42, 0.45, 0.45	0.51, 0.37, 0.43, 0.55, 0.53	0.36, 0.25, 0.29, 0.46, 0.44	0.31, 0.48, 0.35, 0.53, 0.54	0.52, **0.59**, 0.55, 0.58, 0.58	**0.62**, 0.56, **0.59**, **0.63**, **0.62**
Brasilia	0.56, 0.56, 0.56, 0.50, 0.49	0.81, 0.67, 0.73, 0.73, 0.78	0.69, **0.88**, 0.77, 0.71, 0.68	0.75, 0.61, 0.64, 0.63, 0.68	0.84, 0.86, **0.85**, **0.83**, **0.87**	**0.83**, 0.86, 0.84, 0.82, 0.83
Buenos Aires	0.78, 0.76, 0.77, 0.63, 0.42	0.82, 0.99, 0.89, 0.81, 0.67	0.84, 0.92, 0.88, 0.79, 0.57	0.81, **1.00**, **0.90**, 0.81, **0.76**	**0.87**, 0.91, 0.89, **0.82**, 0.70	0.84, 0.90, 0.87, 0.78, 0.73
Medellin	0.57, 0.49, 0.52, 0.60, 0.59	0.73, 0.53, 0.61, 0.69, 0.72	0.67, **0.71**, 0.69, 0.70, 0.70	0.78, 0.57, 0.64, 0.71, **0.81**	0.82, **0.71**, **0.76**, **0.79**, 0.80	**0.89**, 0.53, 0.66, 0.75, 0.80
**Country (↓)**						
Argentina	0.45, 0.45, 0.45, 0.49, 0.49	0.82, 0.70, 0.75, 0.79, 0.80	0.71, **0.81**, 0.76, 0.76, 0.76	0.82, 0.63, 0.67, 0.73, 0.81	0.84, 0.75, 0.79, 0.81, 0.86	**0.91**, 0.74, **0.81**, **0.84**, **0.87**
Brazil	0.54, 0.53, 0.53, 0.50, 0.50	**0.87**, 0.63, 0.73, **0.75**, 0.77	0.36, 0.40, 0.37, 0.29, 0.28	0.66, **0.73**, 0.63, 0.60, 0.73	0.84, 0.65, 0.73, 0.74, **0.80**	0.85, 0.65, **0.74**, **0.75**, **0.80**
Colombia	0.31, 0.32, 0.31, 0.53, 0.48	0.79, 0.65, 0.71, 0.82, 0.82	0.74, **0.72**, 0.73, 0.82, 0.80	0.74, 0.35, 0.39, 0.72, 0.82	0.84, 0.69, **0.76**, **0.85**, **0.87**	**0.86**, 0.68, **0.76**, **0.85**, 0.85

Average precision (Prc.), recall (Rec.), F1 scores, accuracy (Acc.), and area under the ROC curve (AUC) are reported. The bold values represent the best performance for the respective performance measure. For the MANSA dataset, EPIAL and its variant are applied with a history of 4 days, lead time of 3 days, and context window of size 10. As for the LA protest dataset, city- and country-level data are used with a history of 5 days, a lead time of one day, and a context window of size 10.

### 4.5. Model performance with parameter variation

**Figure 4:**
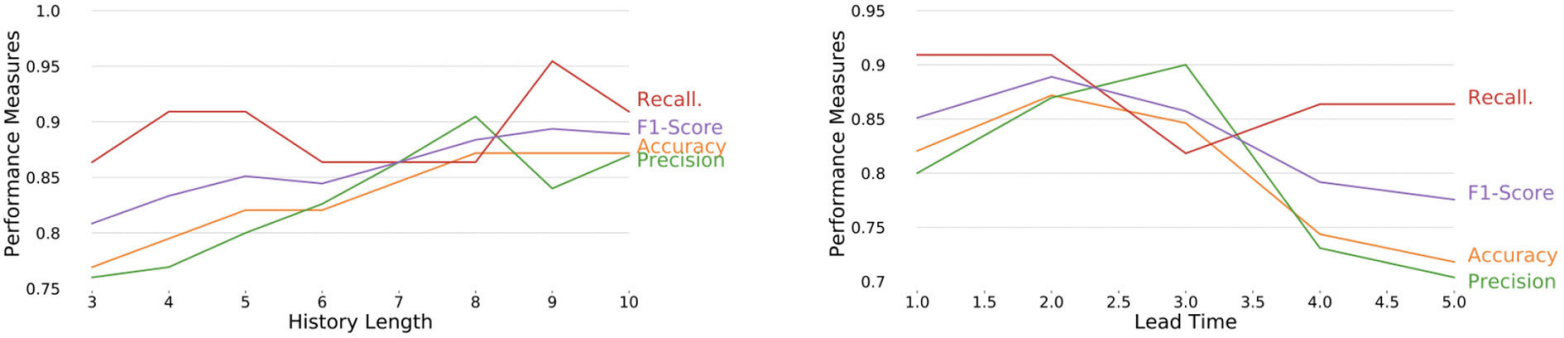
Performance measures of EPIAL over various history lengths **(left**), and lead times **(right)** for the city of Brasilia with a context window size of 10.

We vary important parameters of EPIAL and assess the effects of these parameters on the proposed EPIAL's performance. Some of the key parameters associated with the model are history length, lead time, context window, input dropout rate, and batch size. We observe that model performance shows an upward trend with the increase of history length followed by a downward trend (Figure 4, **left**) for the city of Brasilia. From the perspective of lead time, the model shows a downward trend (Figure 4, **right**) for the city of Brasilia, which is expected as it becomes harder to forecast with the increase in lead time. We also observe similar behavior in terms of history length and lead time for the city of Damascus (refer to Supplementary Figure S3). We present our analyses with other parameters such as context window, input dropout rate, and batch size (refer to Supplementary Figures S4, S5).

### 4.6. Evaluation of precursors

A key capability of the proposed EPIAL methods is that it organically identifies precursors from input articles which could be considered as evidence for the forecast. In this experiment, we identify text excerpts for each document given some predefined keywords, and we then merge overlapping excerpts to form disjoint excerpts for each document. This setting could be thought of as a semi-supervised setting for precursor identification. The model is also capable of taking words of the entire document as input, but it increases the training time and memory requirement. It is also possible to divide each document into equal size excerpts without using predefined keywords.

#### 4.6.1. Quantitative analysis

**Figure 5:**
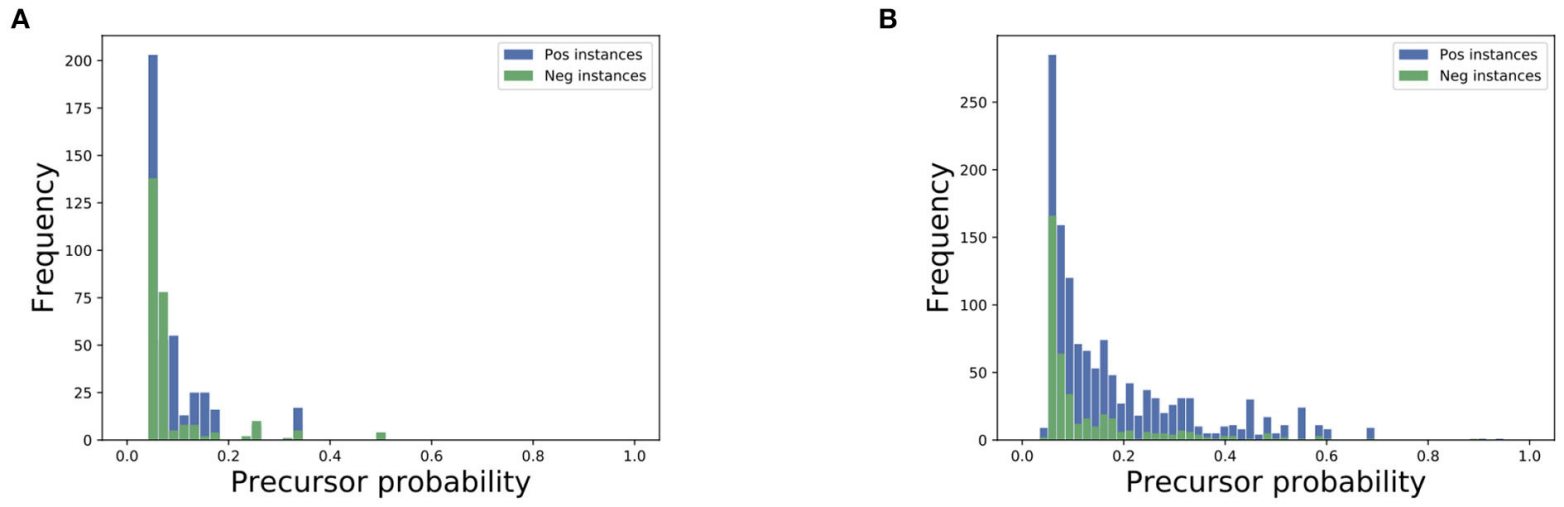
Distribution of precursor probabilities for negative examples (green) and positive examples (blue) for the city of Ar Raqqah **(A)** and Brasilia **(B)**.

For quantitative analysis of precursors, we assess the probabilities assigned to each precursor for positive and negative instances in a dataset, and plot the distributions for positive and negative instances (see Figure 5). We observe that precursors in positive instances have more probabilities assigned compared to the precursors in negative instances. As precursors within positive instances are related to an actual event, these precursors would be on more specific topics, whereas precursors with negative instances would cover more generic topics. As such the precursors with positive instances will tend to have more probabilities assigned compared to the precursors with negative instances.

#### 4.6.2. Qualitative analysis

The attention mechanism in EPIAL and EPIAL-light assigns probability scores to excerpts (or articles). As EPIAL-light only identifies precursors at the document level, here we only qualitatively evaluate the precursors identified by EPIAL, which is capable of identifying precursors both at document excerpts and document levels. The excerpts with higher probability could be considered as precursors to the event in consideration. As the excerpts are in Arabic for the MANSA dataset, and in Spanish or Portuguese for the LA protest dataset in our study, we use Google translate for interpreting the text in English. We also take help from our in-house Arabic expert for comprehending the Arabic precursors.

We observe that the precursors identified by EPIAL are relevant to the events reported in the ground truth. Table 3 shows precursors (Arabic translations) related to the fall of Tal Afar city by ISIS and the conflicts between ISIS and the Syrian democratic force. Table 3 represent precursors (Arabic translations) related to upcoming conflicts near the city of Damascus: a missile hit a site near the Damascus airport on 27 April 2017 and 2 days later, an armed conflict occurred in a location called Assem, which is near Damascus. This development shows the advancement of the rebel group. Table 3 shows two different sets of precursors (Portuguese translations) indicating the event, which is a protest against the corruption of the petrobras, a Brazilian petroleum corporations. Finally, Table 3 shows precursors (Spanish translations) related to a demonstration happened in Argentine capital Buenos Aires. We reported additional case studies in Supplementary Material (refer to Supplementary Section S4).

**Table 3:** Examples of anticipated events.

	**Date**	**News Excerpts (Precursors) [translated Arabic, Spanish, and Portuguese texts are shown]**
Case Study 1	2017-07-05	A desperate sector of young people to feel the frustration of hopeless despair even to the camp of Sias Fadash still dominate other areas such as Tal Afar (**Prob. 0.33**)
wall of the wall of democracy as a result of bombing battles Bombing a jihadist against an element of the organization advocated killed battles Result of a coalition of countries in a month attack supporting the coalition of countries Garo Joe (**Prob. 0.05**)
Case Study 2	2017-04-27	Media intelligence sources revealed a new information hit a missile targeted the sites ofAskarnear the airport Damascus, east ofAsimFajrKhamis (**Prob. 0.47**)
Security force (state actor) [Reported by Al Masdar]
Case Study 3	2014-11-10	Luiz figueiredoa launch init amaraty server promote an act protest delay in rent residence functional diplomat brazilian act in (**Prob. 0.51**)
Case Study 4	2014-06-09	number reach 300 person militant girl give present be vice (**Prob. 1.0**)
claim suspension dismissal occur medium strong retraction sale production sector. (**Prob. 0.45**)
uba be contain nourish operative security police infantry avoid march toward home province good air locate callao (**Prob. 0.55**)

Note that we rely on Google Translate to decipher the meaning of the identified precursors. As the quality of the translation is not guaranteed, we need additional validation strategies. For example, A domain expert will be able to better translate the meaning of the precursors. Another approach could be performing an ablation study. In an ablation study, we could remove the attention network to see the effectiveness of the attention component. We plan to explore this direction in the future.

## 5. Conclusion

Our proposed method EPIAL and its variant offer a unified framework for event forecasts and precursor identification with deep-learning techniques. The methods are able to learn complex relationships between external data sources (e.g., news articles) and events, and are robust with respect to missing external data. The EPIAL model exploits the document at the word level and identifies excerpts within articles for better interpretability using the attention mechanism. Both methods exhibit superiority over recently introduced methods such as MIL and nMIL in terms of predictive capability over two real-world large event datasets at city and country levels. The results show that it is useful to go from the document level to the finer-grain level (document excerpts) for better forecasting and precise identification of precursors. The method is quite robust with parameter variations and scalable to handle different types and sizes of external signals.

In this study, the models predict the only presence of an event. We plan to extend the models to predict multiple events per timestamp and address relevant questions in this direction: which kind of events will happen? which group of events will happen together? We can also discriminate between events in terms of frequency and impact while training the models. It would be interesting to study how fake news affects our models? While creating examples for the models, we take news articles related to a city or country. As such, these models do not explicitly take into account more fine-grained geospatial features—e.g., multiple actors are operating at different influence levels in the middle east. A possible extension of the proposed method would explicitly handle spatial features and forecast events with corresponding geolocation and/or entities involved in the events, thus transforming the models from a coarse-grain to a finer-grain event detection system. Note that the precursors identified by the proposed method could be leveraged by other event and entity extraction methods, e.g., for generating richer event warnings with identified entities in precursors. Finally, it will be interesting to use other data sources in addition to news articles, both textual (e.g., Twitter) and non-textual.

## Data availability statement

Publicly available datasets were analyzed in this study. This data can be found at: https://github.com/planetmercury/mercury-challenge.

## Author contributions

KH, HH, and AG study conception and design and draft manuscript preparation. BK data collection. KH, HH, and YN experimentation. KH, YN, NR, and AG analysis and interpretation of results. All authors reviewed the results and approved the final version of the manuscript.

## Funding

This research is based upon a study supported by the Office of the Director of National Intelligence (ODNI), Intelligence Advanced Research Projects Activity (IARPA).

## Conflict of interest

The authors declare that the research was conducted in the absence of any commercial or financial relationships that could be construed as a potential conflict of interest.

## Publisher's note

All claims expressed in this article are solely those of the authors and do not necessarily represent those of their affiliated organizations, or those of the publisher, the editors and the reviewers. Any product that may be evaluated in this article, or claim that may be made by its manufacturer, is not guaranteed or endorsed by the publisher.

## Author disclaimer

The views and conclusions contained herein are those of the authors and should not be interpreted as necessarily representing the official policies or endorsements, either expressed or implied, of the ODNI, IARPA, or the U.S. Government. The U.S. Government is authorized to reproduce and distribute reprints for Governmental purposes notwithstanding any copyright annotation thereon.
