# Supplementary material for: Identifying geopolitical event precursors using attention-based LSTMs
Source: Front Artif Intell. 2022 Oct 31;5:893875. doi: 10.3389/frai.2022.893875 (PMC9662789; doi:10.3389/frai.2022.893875)
Supplement: Supplementary file 1 [file Data_Sheet_1.PDF]

Supplementary Material

In this supplementary material, we describe methods and analyses that we could not discuss in detail in the main text.

1 METHODS

Our proposed framework for precursor identification (see Sec. 3 and Fig. 2) is based on a two-layer LSTM predictive model. Fig. S1 presents the basic two-layers LSTM model for prediction where we concatenate the documents of each day and learn a representation for the documents for each day: $\mathcal{D}^{(t)} = [D_1^{(t)}; D_2^{(t)}; \ldots; D_{k_t}^{(t)}]$. The first layer of LSTM layer is used to construct a representation for $\mathcal{D}^{(t)}$: $d_t = \mathrm{LSTM}(\mathcal{D}^{(t)})$. We use weight sharing across the LSTM layers of different days. In this framework, $\tanh$ function is used as the activation function in the LSTM layers. After having a representation d_t for the data of each day, the second LSTM layer is designed to take the sequence $d_{i}{}_{t=t-h+1}^{t}$ as input and provides a probability that an event will happen: $\widehat{y} = \mathrm{LSTM}(d)$. We use sigmoid activation function in this final LSTM layer.

We extend this model (see Fig. S1) by adding attention mechanism between two LSTM layers for identifying precursors. For this, instead of concatenating documents per day we keep the documents separate while giving input to the first LSTM network and learn representation for each of the document. The learned representation are given as input to an attention network (see Fig.2b–2c)

2 EARLY STOPPING IN TRAINING MODELS

While training the model we use early stopping criteria as show Fig. S2. For model selection we identify a strip of epochs where validation loss is stable and we select the epoch for which the average validation loss of its neighboring epochs is minimal.

3 STUDY OF MODEL PARAMETERS

We perform analyses of EPIAL's performance with varying parameters such as history length, lead time, context window, input dropout rate and batch size. We observe that model performance shows upward trend with the increase of history length followed by a downward trend (Fig. S3(left)) for the city of Damascus. From the perspective of lead time, the model shows downward trend (Fig. S3(right) for the city of Damascus, which is expected as it is harder to forecast with the increase of lead time. With context window, we observe a mix of upward and downward trend for Ar Raqqah and slightly upward trend for Brasilia (Fig. S4). For both input dropout rate and batch size, the model exhibits upward trend followed by a downward trend (Fig. S5).

4 QUALITATIVE ANALYSIS

The attention mechanism in EPIAL assigns probability scores to each excerpts (or articles). The excerpts with higher probability could be considered as precursors to the event in consideration. As the excerpts are in Arabic, Spanish, or Portuguese for the study, we use Google translate for interpreting the text in English. We also take help from our in-house Arabic expert for comprehending the Arabic precursors. We observe

precursors identified by EPIAL are relevant to the events reported in the ground truth. Casestudy 1–2 represent precursors (Arabic translations) related to the city of Ar Raqqah and Damascus which indicates some of the upcoming conflicts in the region. Casestudy 3 shows a set of precursors (Portuguese translations) indicating the event, which is a protest against the corruption of the petrobras, a Brazilian petroleum corporations. Finally, Casestudy 4 shows precursors (Spanish translations) related to a demonstration happened in Argentine capital Buenos Aires.

4.1 Figures

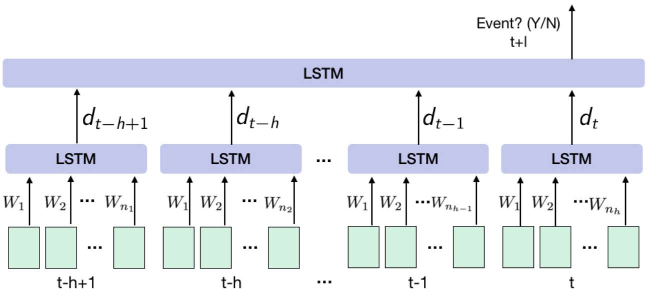

Figure S1. Event prediction using a two-layer LSTM model.

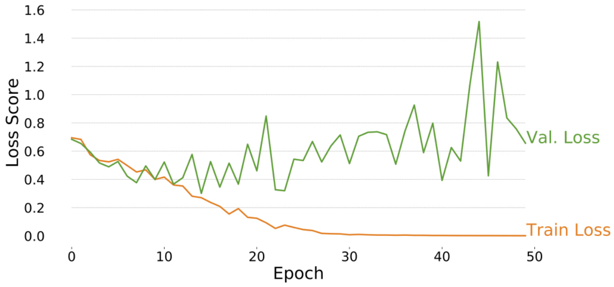

Figure S2. Stopping criteria: observe the train and validation loss and identify a strip where validation loss is stable and not very divergent form the train loss. Here in this figure we can pick the model at epoch 15 from the strip of epochs 7 to 18.

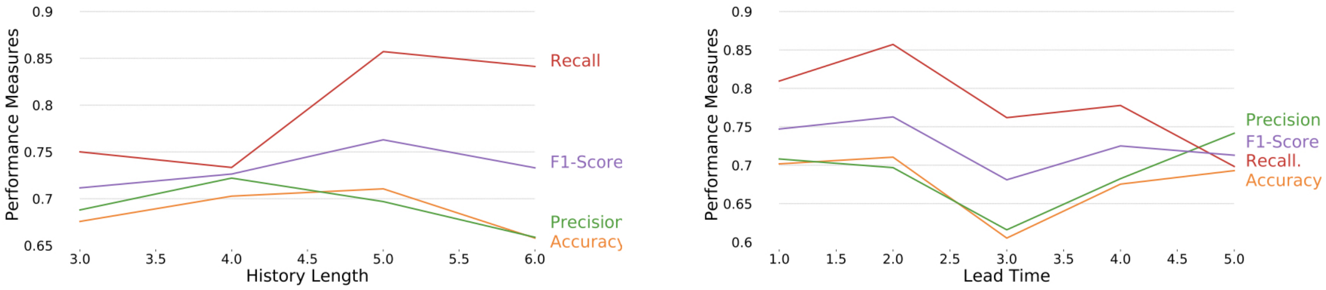

Figure S3. Performance measures of EPIAL over various history length (left) and lead time (right) for the city of Damascus for test data context window of size 10.

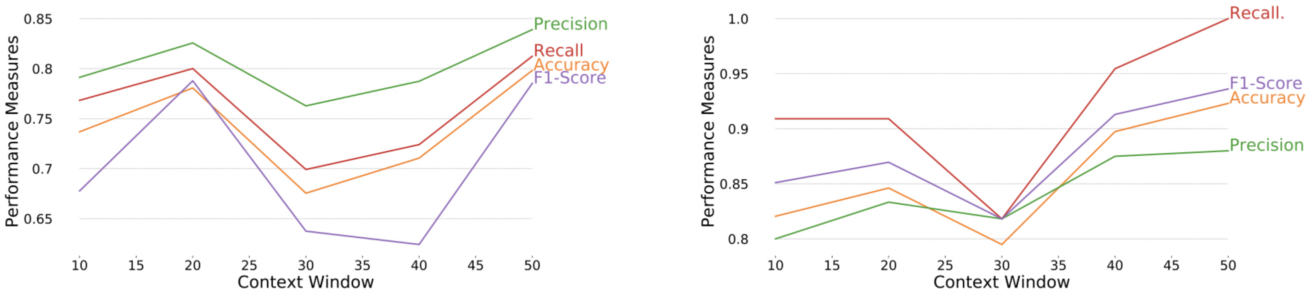

(a) Ar Raqqah (b) Brasilia

Figure S4. Performance measures of EPIAL over various context windows for the city of Ar Raqqah (left) and Brasilia (right) for test data with history of 5 days and lead time of 2 days.

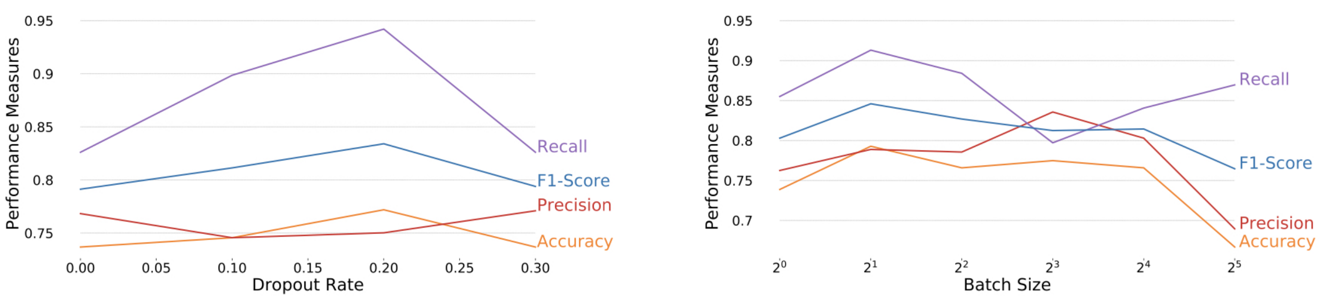

(a) Ar Raqqah (b) Ar Raqqah

Figure S5. Performance measures of EPIAL over various LSTM input dropout rates (left), and batch sizes (right) for the city of Ar Raqqah for test data with history of 4 days and lead time of 3 days.

	Date	**News Excerpts (Precursors)** [Translated Arabic, Spanish, and Portuguese Texts are shown]
Case Study 1	2017-03-21	Will lead to real disasters that would harm American interests, pushing about 1,000 American soldiers north of the fence in preparation for a battle that is not proof of existence (**Prob. 0.49**)
		Papers have been released without cover to protect them. Everyone knows that an armed organization has been declared a moderate opponent and is only a total of terrorism (**Prob. 0.5**)
	2017-03-22	Mshirf Karmouh spoke Syrian media Twitter site Download a soldier time Bdgu Syria democracy by the transfer of soldiers near Yousra fled near the Ja39;far Quarry board of warships (**Prob. 0.17**)
		It is used as a displaced product near the town of Tabak Rural Park (**Prob. 0.17**)
		Gharat Mansur villages of the vicinity of the most active active showcased a real garde much higher shown to see the retrieved bodies of the arrival of the organization (**Prob. 0.16**)
		**Matched Ground Truth Event**
	2017-03-27	Location: Near Raqqah; description: SDF launces an attack on ISIS.
Case Study 2	2017-04-19	Sur Beshn attack denied the head of the Bashar Assad, the official army of Garrah Khan Sheikh accused Western countries subject to justify the strike of a US missile targeting the airport Shaker Askar, Apr 7. (**Prob. 0.95**)
	2017-04-21	An opposition official with a civilian gunman fighting a country besieged by Syria has drawn up the framework of an exchange deal, media said (**Prob. 0.14**)
		**Matched Ground Truth Event**
	2017-04-25	Location: Outskirts of Damascus; description: Syrian Arab Military targeted unspecified non-state actor
Case Study 3	2014-07-10	Union movement housing front fight housing hold in evening fifth feiraran act in repudiation violence abuse police commit lawyer beneditorobertobarbosaon last day 25june one (**Prob. 0.26**)
		A protest carry out national union moedeirosinterdict partial lane copacabana beach flamengoin afternoon quinta feiragroup form about 100 person ask reform plan cargo (**Prob. 0.26**)
		Diverse activist representative comiso human right participate a protest in front bar (**Prob. 0.23**)
		Worker at work petrochemical complex rio janeiro make demonstration in quintafeira highway(**Prob. 0.24**)
	2014-07-12	Ten person meet in road city place where to have start protest start (**Prob. 0.55**)
	2014-07-13	Or stop Brazil near 50 demonstrator gather in square three power in Brazil (**Prob. 0.53**)
		Police officer disperse with use bomb gas moral effect a protest canopy prison activist in operation civil police act happen in plaza saenspenazonar north with participation about thousand person intend to follow towards stadiummaracana but to be to prevent a strong scheme security situation tense with police officer (**Prob. 0.47**)
	2014-07-14	Integral movement worker without ceiling cearamtstceara block 2 hour (**Prob. 0.14**)
		About ten brazilian make a small demonstration in front copacabana palace observe several foreign they sing next refrain prisoner political freedom already fight not be crime you go in (**Prob. 0.66**)
		**Matched Ground Truth Event**
	2014-11-15	Location: Brasilia; description: In the federal capital, about 800 people perform an act in the afternoon of this Saturday in front of the national congress, in Brasilia, in protest against the report of corruption in Petrobras.
Case Study 4	2017-04-19	above only argentina 77 point venezuela 20 point agreement with communiqué disclose fgv ifo fall in Brazilian indicator below average(**Prob.**)
	2014-02-17	see ecuador quito ecuadorian president rafael belt ask monday support reelection official august barrier mayor quito avoid occur venezuela argentina bolivia according to say opposition enquer capital try to destabilize national government progressive struggle political look municipal election next Sunday note capital ecuatoriano mayor official barrier movement alliance country aspire reelection mauricio roda opponent create create opportunity symbolize polarization electorate Quito belt secure elections municipal capital game stability political country call citizen support barrier continue process revolution citizen denominate strategy government president radial interview recognize monday to occur say step progressive government venezuela bolivia argentina belt if roda succeed being able to produce similar situation venezuela day president nicolas maduro have own opposition caracas argentina m andataria cristina fernandez day have claim protest capital right hand revolution citizen game be tactical have used venezuela argentina bolivia determine city enquistar local government go destabilize national government progressive left that be tactic right insist president(**Prob. 0.24**)
		**Matched Ground Truth Event**
	2014-02-18	The kirchnerist group integrate and organize to meet this afternoon in front of the embassy of Venezuela in Argentina to express their support for the Bolivarian revolution and the government of President Nicolas Maduro
